# The Regulation of Gonadal Somatic Cell Differentiation in Humans

**DOI:** 10.1016/j.gpb.2022.04.003

**Published:** 2022-04-30

**Authors:** Min Chen, Fei Gao

**Affiliations:** 1 Guangdong and Shenzhen Key Laboratory of Male Reproductive Medicine and Genetics, Institute of Urology, Peking University Shenzhen Hospital, Shenzhen Peking University-The Hong Kong University of Science and Technology Medical Center, Shenzhen 518036, China; 2 State Key Laboratory of Stem Cell and Reproductive Biology, Institute of Zoology, Chinese Academy of Sciences, Beijing 100101, China; 3 Institute for Stem Cell and Regeneration, Chinese Academy of Sciences, Beijing 100101, China; 4 Beijing Institute for Stem Cell and Regenerative Medicine, Beijing 100101, China; 5 University of Chinese Academy of Sciences, Beijing 100049, China

In mammals, both male and female gonads are derived from the bipotential gonadal primordium, *i.e.*, the genital ridge. Primordial germ cells (PGCs) are the germ cell progenitors that arise from the extraembryonic ectoderm, migrate through the hindgut endoderm, and reach the genital ridge before sex determination [1]. Both Sertoli cells and granulosa cells are derived from somatic cells in undifferentiated genital ridges. The differentiation of germ cells and gonadal somatic cells during gonad development is regulated by various factors, including transcription factors, epigenetic regulators, and environmental factors. The interactions between germ cells and somatic cells also play critical roles in gonad development and PGC differentiation. The differentiation of PGCs is affected by the signals derived from the surrounding somatic environment, indicating that the surrounding gonadal microenvironments may tightly regulate the behaviors of PGCs [2], [3], [4]. The regulation of PGC and gonadal somatic cell development in mouse models has been extensively investigated previously [5], [6], [7]. Several research groups have described the developmental transcriptomic landscapes of human fetal germ cells (FGCs) and spermatogenesis, with studies mainly focusing on the development of germ cells [8], [9], [10], [11], [12], [13], [14], [15], [16], [17]. A variety of germ cell types and states have been well demonstrated throughout the development in human fetal, neonatal, infant, and adult stages [9], [11], [12], [18]. However, the regulation of gonadal somatic cell differentiation and the interactions between germ cells and gonadal somatic cells in humans still remain incompletely understood.

Recently, Wang et al. [19] systematically analyzed the fetal germ cells (FGCs) and gonadal somatic cells in human embryos and fetuses using a time-series single-cell RNA sequencing (scRNA-seq) strategy. They also analyzed the development of both germ cells and gonadal somatic cells in a Turner syndrome embryo (45, XO). This study is the first to systematically analyze the gene expression patterns and cell type compositions in monosomy X (45, XO) gonads compared with normal female (46, XX) and male (46, XY) embryos at the same developmental stage (7 weeks of gestation; 7 W). The results suggest that the lack of one copy of X chromosome has different influences on germ cells and gonadal somatic cells. It is proposed that the XO embryo is likely originated from an XX zygote (specifically losing one copy of X chromosome). X chromosome inactivation (XCI) is an essential mechanism to compensate for different dosages of the X-linked genes between female (XX) and male (XY) cells. After specification, germ cells in normal female gonads reactivate the inactivated X chromosome, which will remain active during FGC development. On the other hand, in the gonadal somatic cells, the inactivated X chromosome will be maintained at the inactivated state permanently. Therefore, X chromosome monosomy would cause different outcomes in germ cells and somatic cells. In germ cells, it leads to the loss of one active X chromosome (all of the genes on the active X chromosome are ‘active’, permitting being expressed). However, in gonadal somatic cells, it leads to the loss of one inactive X chromosome (majority of the genes on the inactive X chromosome are epigenetically silenced with only dozens of genes escaping the inactivation process and keeping being actively expressed). This may partially explain the cell type-specific abnormalities in the gonads of Turner syndrome embryos. Furthermore, X chromosome monosomy leads to the depletion of Purkinje cell protein 4-positive and tachykinin precursor 1-negative (PCP4^+^TAC1^−^) cell type in the Turner syndrome embryo. Alternatively, if the XO embryo is originated from an XY zygote (specifically losing Y chromosome), the phenotypes would suggest that the genes on Y chromosome are not essential for the development of germ cells and some types of gonadal somatic cells.

In this study, Wang et al. [19] validated the presence of some abnormal cells that simultaneously expressed classical markers of gonadal somatic cells and germ cells at three different developmental stages (8 W, 16 W, and 21 W) with three different combinations of antibodies, POU class 5 homeobox 1/delta like non-canonical Notch ligand 1 (POU5F1/DLK1), synaptonemal complex protein 3/WT1 transcription factor (SCP3/WT1), and DEAD-box helicase/forkhead box L2 (DDX4/FOXL2) [19], which has not been reported previously. What will be the final fate of these abnormal cells? Will these abnormal cells gradually disappear with development, or will they develop into some teratoma ‘progenitor’ cells in the adult? Follow-up investigations are needed to answer these interesting questions.

In addition, Wang et al. [19] also identified a new subtype of germ cells that highly expressed secreted protein acidic and rich in cysteine (*SPARC*), a master gene regulating cell migration. According to previous studies, germ cell migration occurs from the hindgut along the gut and across to dorsal mesentery to reach gonads at around 4–5 W in humans, thus most of the germ cells have already completed the migration process at around 5 W. However, the SPARC^+^ germ cells can be found in the gonads of human fetuses up to 23 W, suggesting that after entering the gonad, some of the germ cells may still maintain the migration potential and could migrate within the gonad to optimize their spatial distributions there.

Wang et al. [19] have found that the bone morphogenetic protein (BMP) signaling pathway has developmental stage-dependent functions for male germ cells, and the BMP signaling activity specifically promotes the expression of *ALDH1A2* [a gene encoding a retinoic acid (RA)-synthesizing enzyme] in male germ cells at relatively late developmental stage (15 W). Further studies have demonstrated that the BMP signaling pathway plays vital roles in the gonocyte-to-spermatogonium transition (GST) process.

This study has also revealed the functional crosstalk between the BMP signaling pathway and RA signaling pathway in male gonadal somatic cells. BMP signaling activity inhibits the expression of *ALDH1A3* (a gene encoding a RA-synthesizing enzyme) in all testicular somatic cell types, Sertoli cells, Leydig cells, and keratin 19-positive (KRT19^+^) cells, at both the early (7 W) and late (15 W) developmental stages. Notably, different gonadal somatic cells exhibit different responses to BMP inhibition. BMP and RA signaling pathways play critical roles in regulating the meiosis of germ cells [6], [20], [21], [22]. These results would provide important information for better understanding the regulation of meiosis initiation in germ cells.

Furthermore, Wang et al. [19] have also assessed the developmental origins of the granulosa cells in females and those of the steroidogenic lineages (Leydig cells in males and theca cells in females) in both genders. The results indicate that DLK1^+^ cells in early developmental stages of male and female embryos may further differentiate into Leydig cells in testes and theca cells in ovaries. *DLK1* was continually expressed in Leydig cells of the testes, while its expression disappeared in theca-like cells of ovaries. The origin of steroidogenic cells is a controversial question with a lot of debates in the literature. The results indicate that DLK1^+^ cells are probably a new type of progenitors of steroidogenic cells. They have also proposed that TAC1^+^ cells at 7 W may further differentiate into granulosa cells in later development stages. However, these conclusions need to be validated by lineage tracing experiments in other animal models.

Finally, Wang et al. [19] have found that Sertoli cells, rather than Leydig cells, express *HSD17B3* during fetal and neonatal periods. Leydig cells do not express *HSD17B3* until neonatal stages and the ratio of cells that express *HSD17B3* gradually increases afterward. These results indicate that the androstenedione is produced by Sertoli cells, but not by fetal Leydig cells during fetal stages, which is consistent with the findings in mouse model [23], [24], [25], suggesting that the regulation of steroidogenesis in fetal male gonads is conserved in mice and humans.

Together, Wang et al. [19] have systematically analyzed the regulation of FGC and gonadal somatic cell development in humans. They have identified and characterized new types of FGCs and gonadal somatic cells, and verified all of them by systematic immunofluorescent staining. They have proposed that the DLK1^+^ cell population is a progenitor population of the steroidogenic cell lineage and the TAC1^+^ cell population is a progenitor population of granulosa cells (Figure 1). Notably, they have demonstrated the crosstalk between BMP and RA signaling pathways by functional assays. The single-cell omics studies are greatly helping advancing the human developmental biology, filling the gaps between the functional studies in mouse model and cell atlas studies in humans. This study provides a complex but highly ordered development and interaction network for the human FGCs and gonadal cells.

**Figure 1:**
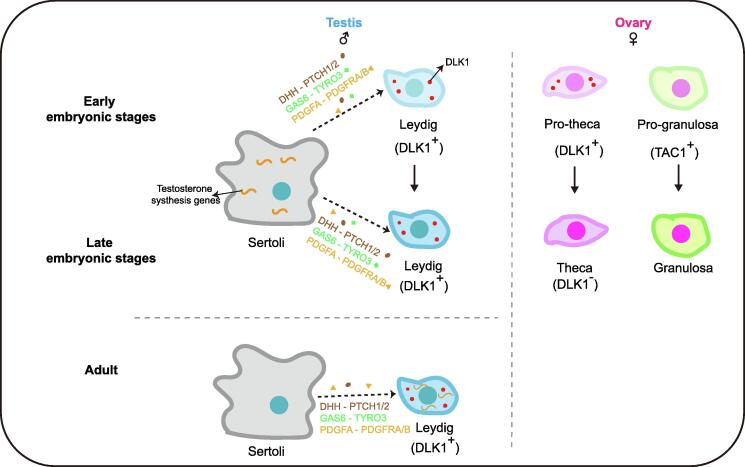
**The regulation of gonadal somatic cell differentiation in humans**

## CRediT author statement

**Min Chen:** Writing ‐ original draft, Writing ‐ review & editing. **Fei Gao:** Funding acquisition, Writing ‐ original draft, Writing ‐ review & editing. Both authors have read and approved the final manuscript.

## Competing interests

The authors have declared no competing interests.
